# The “Our Voice” Method: Participatory Action Citizen Science Research to Advance Behavioral Health and Health Equity Outcomes

**DOI:** 10.3390/ijerph192214773

**Published:** 2022-11-10

**Authors:** Maja Pedersen, Grace E. R. Wood, Praveena K. Fernes, Lisa Goldman Rosas, Ann Banchoff, Abby C. King

**Affiliations:** 1 Department of Epidemiology and Population Health, Stanford University School of Medicine, Stanford University, 1701 Page Mill Road, Palo Alto, CA 94304, USA; 2 School of Sport, Exercise and Rehabilitation Sciences, University of Birmingham, Edgbaston, Birmingham B15 2TT, UK; 3 Department of Health Services and Policy, Faculty of Public Health and Policy, The London School of Hygiene and Tropical Medicine, Keppel Street, London WC1H 9SH, UK

**Keywords:** participatory research, community-engaged research, citizen science, health equity, health promotion, community health, food security, physical activity

## Abstract

Citizen science research that more fully engages the community can systematically involve people from under-resourced groups to create practical health-enhancing improvements across physical, social and food environments. Exemplary health equity-focused outcomes include key health behaviors (e.g., healthy eating or physical activity) and community-level changes (e.g., public transit to food shops) that are central to health promotion while being demonstrably impacted by local environmental contexts. Yet, few examples of this approach are readily available for application within complex, community-based settings. In this paper, we present the *Our Voice* (OV) four-step method to demonstrate an integrated participatory citizen science approach and its usability for action-focused researchers and community health practitioners. In addition, we present a summary of the major research, processes, and community outcomes, with examples drawn from nutrition and healthy food access areas, among others. Finally, we explore the hallmark features of the OV method that effectively engage citizen scientists, empowering action and fostering solution-building across social and environmental structures impacting community health. Expanding research that marries participatory research philosophies with innovative citizen science methods, supported by systematic data collection, visualization, and delivery technologies, in turn provides a powerful toolkit for tackling local to global health equity challenges.

## 1. Introduction

Health equity, defined as a state where “everyone can attain their full potential for health and well-being” [1] (p. 1), has increasingly become a major goal of global public health efforts. Achieving health equity demands a process of systematically identifying and reducing unfair, avoidable, or remediable differences within and among groups of people. As such, health equity promotion efforts generally strive to design and distribute resources, policies, and programs most likely to help equalize health outcomes across under-resourced social groups and their more resourced counterparts [2]. It stands to reason, then, that innovative health equity strategies must amplify and center the voices of groups experiencing health inequities, embedding their collective perspectives in decision-making processes and policies that impact community development from local to global spheres of influence.

Participatory approaches to health and social research aim to address the impacts of historical injustices and equalize power dynamics between researchers and non-researchers, and to actively engage communities that stand to be most directly impacted by the research findings, in the research process [3]. This research philosophy has its roots in Brazilian educator Paulo Freire’s theory of critical consciousness, which posits that participants who critically reflect on their lived reality build the foundations of empowerment and the potential to create local solutions that challenge the structures of their own oppression [4]. Such engagement in research is intended to optimize the relevance and feasibility of research findings, thus maximizing impact [5]. While employing a variety of methods and representing diverse research questions, documented participatory approaches—including participatory action research, co-production, integrated knowledge translation, community-engaged research, and community-based participatory research—are situated along an intersecting continuum of research and advocacy and generally converge on the common aim of co-creation of knowledge resulting from both researcher and participant expertise [5,6,7,8,9]. Together, these approaches represent powerful practices for inclusion of groups experiencing a disproportionate burden of death and disease in research that can lead to practical improvements across relevant health indicators [9], including improvements in key health behaviors (i.e., healthy eating, physical activity, tobacco use) that are central to health promotion while being demonstrably impacted by local environmental context.

Citizen science is a particularly promising approach for engaging lay (i.e., non-research) members of the public as active agents in the research process. This approach is rooted in residents’ contextual knowledge generated outside of formal scientific institutions [10], which can lead to new types of scientific knowledge, advocacy action, and/or policy change [11]. Traditional definitions of “citizen” often are used with this approach to signal community resident participation regardless of legal citizenship status. Although citizen science has a lengthy history of application within the natural and environmental sciences, more recent adaptations have focused on social, biomedical, and public health issues, leading to a growing scientific literature base [12,13,14]. Citizen science methods have not been as widely applied within the field of public health, yet they show promise for addressing health equity issues and the potential for democratizing access to scientific knowledge by diverse groups in addition to aiding real-world translation of knowledge into meaningful action [15].

Pairing participatory practices with citizen science research methodology creates new synergies for innovative, action-oriented research to promote health equity. This approach focuses on community residents as ideal agents of local change, fusing the action orientation of participatory research with the innovative data collection methods, data visualizations, delivery, and community-driven outputs of citizen science methodology. However, few examples of this approach are readily available and accessible for application within complex, community-based settings.

The purpose of this paper is to describe one form of this type of promising participatory scientific approach, the *Our Voice* (OV) citizen science research method, with an emphasis on identifying the outcomes and hallmark characteristics of this approach that engages the voices of under-resourced groups to expand decision-making processes for health equity-focused change. This approach is relevant across a variety of sectors, from community residents to researchers, practitioners, community activists, policymakers, planners, and educators. Developed as part of the *Our Voice* Global Citizen Science Research Initiative at Stanford University, the OV method has been widely used across diverse health equity-focused research projects to address numerous community-level challenges affecting diverse and underrepresented groups [16,17]. These applications have yielded a growing evidence base of impacts across different community populations and levels. This evidence base, as well as the OV website, goes over common study practices in partnership building, recruitment, logistics of digital tool use, and data collection and analysis [18]. This paper aims to (1) describe the methods and applications of the OV initiative among action-focused researchers and community health practitioners; (2) summarize outcome categories associated with each step of the method, with a special focus on examples drawn from the nutrition and healthy food access area; and (3) identify hallmark features of the method that effectively engage citizen scientists in a process to empower action and foster solution-building to address social and environmental structures impacting health within their own communities.

## 2. The OV Method for Action-Focused Research

### 2.1. Theoretical Foundations

The OV method is conceptually rooted in the socioecological framework; it features progressive steps to draw out local perspectives and experiences associated with individual, environmental (social, natural, built), and policy domains that impact health [13,19,20]. This approach encompasses the interactive influences of conditions of different community settings (e.g., schools, parks, neighborhoods) with roles and behaviors of individuals and groups, and their joint impacts exerted on health [16,17,20]. Accordingly, the OV method activities and associated outcomes have demonstrated impacts across socioecological levels and domains, from individual behavior change among citizen scientist participants to improved social resources, enhanced environmental features, and informed policy or programmatic decisions for positive change [15,21].

### 2.2. The OV Method

**Figure 1:**
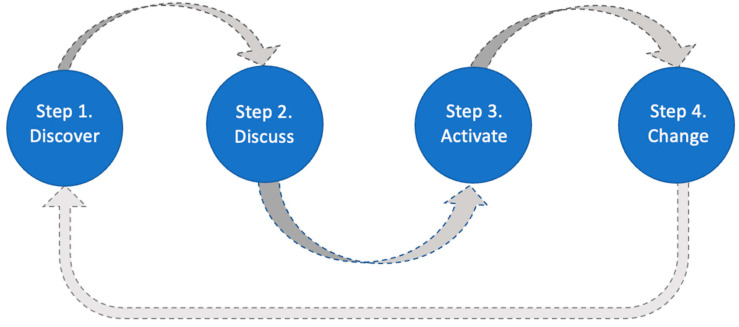
The OV four-step method.

The OV method features four steps [18,22], summarized below and in Figure 1. The OV method is advantageous in comparison to other participatory approaches in that it utilizes and builds upon the foundations of community-based participatory research through its four-step citizen science method. These foundations are strengthened further in its resident-based data collection that effectively engages a range of residents and community stakeholders to direct and produce impactful community changes. These strengths, which have been seen across the global use of the OV method [16,17], are reflected upon further throughout this paper, identifying strengths and impacts that have been produced across research, process, and community outcomes of OV studies.

Step 1: Discover. The Stanford Healthy Neighborhood Discovery Tool mobile app is utilized by citizen scientists to collect data on a community-relevant topic of importance, which is often identified by a community-based group, or through an existing community-based research partnership. The process begins with photographs taken by community members in their local environments. The Discovery Tool app augments photographic data with geotagged maps, audio-textual narratives, and simple positive/negative ratings (via happy- or sad-face emojis) which help in contextualizing the citizen scientist-assigned significance of the photographed features.

Step 2: Discuss. Through facilitated in-person or remote groups, OV citizen scientists subsequently engage in a participatory process to thematically organize the collective data they have generated. They also utilize interpretive and consensus-building processes to strategize ways to build on local assets and address challenges.

Step 3: Activate. Following data analysis, citizen scientists engage in a facilitated participatory process to generate data-driven action plans for community engagement and/or advocacy to address prioritized themes, including identifying the types of relevant stakeholders and decision-makers with whom they could partner.

Step 4: Change. The data-driven action plans form the impetus for change, which can result in a range of modifications to social and environmental features for improved health, as well as longer-term ripple effects affecting additional outcomes after the official research project commences or has ended.

### 2.3. Applications

A growing evidence base characterizes the OV method as an accessible, adaptable, and impactful approach to community-engaged participatory research [16,17], It has been applied to numerous health equity topics, including food environments [23,24], active living and physical activity [25,26], gender-based violence [27], and accessibility in public spaces [28].

Studies have taken place across a range of settings, including urban neighborhoods [29], parks [28], farmers’ markets [30], schools [31], college campuses [27], and rural communities [32]. Likewise, members of diverse population groups have participated as citizen scientists across dozens of OV global research projects spanning six continents, with emphasis on youth [17], older adults [16], low-income communities [33], and other groups historically underrepresented in decision-making processes.

## 3. Research, Process, and Community Outcomes

To summarize the overarching effects associated with the OV method, outcomes are organized into three main categories reflected across the processes and outcomes of OV studies: research, process, and community. Although specific short- and long-term outcomes from studies using the OV method have been detailed elsewhere [15], here we present an overview of the outcome categories to illustrate how the method can build knowledge and advocacy for both professional researchers and citizen scientists at each step (Table 1). Research outcomes include indicators of increased knowledge and are linked to the scientific question underpinning a study, such as experimental factors, or those related to feasibility and/or implementation, and are often of specific interest to research-focused partners. Process outcomes include indicators of increased capacity within and across a partnership and “power building” within the community, and are linked to communication, relationship building, and capacity building among project partners. Community outcomes indicate improvements in community health and represent community-level factors related to health equity. As a package, the summarized outcomes underscore how the OV method is especially suited to action-oriented research approaches. Table 2 highlights specific examples of research, process, and community outcomes in the fields of healthy eating and the food environment.

Below, we provide context to the major outcome categories across Steps 1–4 of the OV method and share examples from applications of the OV method in the literature. Table 2 illustrates examples of research, process and community outcomes related to healthy eating and the food environment.

**Table 2:** Examples of research, process and community outcomes related to healthy eating and the food environment.

Study	Focus	Study Details			Outcomes by Category		
		Location	Citizen Scientists	Metrics	Research (Building Knowledge)	Process (Building Capacity)	Community (Building Health)
Chrisinger et al. (2018) [23]	Healthy Corner Store Network	New Jersey, USA	8 adult community members 18–40 years	Documenting resident perceptions of Healthy Corner Store Network access and food environments	Factors that influence choosing a store included food quality, item selection, sales, and items for special diets Future healthy corner store strategies were identified, such as healthy produce selection, freshness of food, and accessibility	Citizen scientists presented their findings to community leaders and decision-makers Responsible stakeholders were identified to create improvements to corner store access	Findings were used by Healthy Corner Store staff for the next strategic plan, and were embedded into planning documents for a Health Corner Store network
Buman et al. (2015) [30]	Shoppers’ experiences in an urban famer’s market	Southwestern USA	38 adult shoppers aged 18–35	Factors that enhance or diminish the experience of shoppers in an urban farmer’s market	Identification of features that enhance or detract from an urban farmer’s market shopping experience, including: - Product presentation - Interaction with others - Healthfulness of products - Nearby attractions i.e., café - Craft items	N/A	Common features that can guide the farmer’s market to facilitate improved shopper experiences, including: - Freshness - Product presentation - Social interaction - Price of products
Sheats et al. (2017) [24]	Healthy food environments	California, USA	23 older adults (aged 61–92 years)	Factors that impact accessibility, choice, and buying healthy food, and experiences of older adults while navigating food environments	Important features for shopping for groceries were identified, including: - Quality of food items - Cost/pricing promotions - Variety - Foods for special diets - Prepared items Shoppers also visited up to four different stores to compare and find lower prices	Citizen scientists and researchers continued additional advocacy training and developed an advocacy team Relationships were built between citizen scientists, organizational partners, and researchers, creating ripple effects	Citizen scientists: - shared project information with neighbors - Communicated with policymakers to catalyze change - Partnered with other neighborhood groups to address pedestrian safety, presenting implications for public transportation and walkability
Seguin et al. (2015) [32]	Barriers and facilitators to rural healthy eating and active living	New York, USA	24 rural adults (mean age 69.4 years; SD 13.2)	Conducting environmental audits to identify factors that prevent or promote healthy eating and active living.	Use of the Discovery Tool app demonstrated that it was: - Helpful for identifying and prioritizing opportunities for community improvement - Easy, interesting, and fun to use - Self-explanatory	Adults using the Discovery Tool app increased awareness of environmental factors in their communities, cultivating new perspectives on active living and healthy eating	A set of common barrier and facilitator themes to active living and food environments that hold potential for future community change, including: - Walkable destinations such as shops - Restaurants - Non-traditional food stores - Supermarkets

### 3.1. Step 1. Discover

Step 1 research outcomes involve simultaneous identification of both individual-level and environment-level data that reflect outcomes associated with the citizen scientists’ embodied experiences, such as influences, choices, and behaviors in the selected setting [13]. Studies that emphasize distinct citizen scientist populations, such as older adults or school-age children, within specific settings such as neighborhoods or school environments, can advance understanding of the interactive effects of environment and individual experience in those specific populations [31,34]. Evaluation of the Discovery Tool app and process has yielded acceptability and usage information, which have included both positive experiences [31,32] and opportunities for improvement [27].

Step 1 process outcomes feature “power building” in the community as the citizen scientist participants drive the content of the data collection process. Citizen scientists have reported individual benefits related to a sense of ownership over the data they collected, which can improve engagement in the project [26] and heightened awareness and/or a new perspective concerning environmental features related to community health [32].

Step 1 community outcomes can be fostered through citizen scientists’ identification (through photographs, comments, rankings) of social and environmental community features that support or hinder health. Discovery Tool data are integrated into a physical or digital “data package” by the Stanford OV data processing team and can be shared freely with stakeholders and citizen scientists for use in advocacy efforts. The data package contributes local voices to broader, resource-intensive, or systemic issues. For example, the data can underscore the need for better access to fresh fruits and vegetables and inclusive spaces at schools for individuals with special learning needs, or identification of unsafe roads and traffic conditions [31,32].

### 3.2. Step 2. Discuss

Step 2 features facilitated data review and interpretation discussions among participants, which can enrich research findings with context and additional perspectives, advancing understanding of embodied experiences and needs. As participants together review photos, comments, and geotagged locations, group discussions add nuanced perspectives and elicit themes that represent individual and collective experiences [28,33], or elucidate points of tension or disagreement. This can produce research outcomes in which improved understanding of multiple perspectives of resident needs in their direct living environments, guiding researchers to link subsequent steps in the method directly to community needs and priorities [23].

Process outcomes associated with group data review and interpretation include heightened awareness (among citizen scientists) of collective issues across distinct locations (e.g., neighborhoods, schools) and broader community environments, as well as consensus-building processes among group members. Although research team members and community stakeholders are often present at this stage, the emphasis is on citizen scientists discussing their own collective data and generating themes, which enhances transparency of the research process, fostering trust and improved understanding in the partnership [17,35].

The group process can foster community outcomes by creating new linkages and relationships across citizen scientist community members and building understanding between citizen scientists and stakeholders, as well as identifying trusted stakeholders within a community [23]. The group process can also strengthen community connectedness and cohesion through collective solution building and priority setting for action [27,28].

### 3.3. Step 3. Activate

Step 3 research outcomes encompass a group process to thematically prioritize topics raised by citizen scientists, generating a consolidated set of relevant directions for additional research inquiry toward improved understanding or positive change [36]. Creating space for dialogue on priority issues among citizen scientists, stakeholders, and researchers can illuminate shared resources among all actors involved, while leading to further co-produced data and actionable outcomes [28]. Participant advocacy training at this stage can contribute new skills, cultivate empowerment, and disseminate new knowledge about local areas among community members [24,28]. This is facilitated through the opportunity for participants and stakeholders to directly engage in experiences and ideas for solutions [36].

Step 3 process outcomes feature relationships built between citizen scientists and community stakeholders that include the exchange of local knowledge and desired actions, which can help to bridge gaps between service users and providers [28]. For example, a meeting between citizen scientist park users and policymakers provided an opportunity for park users to praise local programming and point out specific aspects critical to the continued success of the program; in response, policymakers stressed the value of this community-delivered, data-driven feedback as an opportunity to clarify information about district-level accountability for factors related to programming and maintenance [36]. Similarly, interest among older adult citizen scientists living in an affordable senior housing site to develop a community garden adjacent to the site led them to seek out information and guidance from local nonprofit organizations with expertise in cultivating and preparing the vegetables that were subsequently grown there by the older adults [25,34].

For Step 3 community outcomes, alongside outcomes at the policy level, data-driven participant advocacy with decision-makers can reinforce support for existing plans or provide guidance to shape or modify planning considerations (e.g., accessibility, inclusiveness) for existing resources or programs [28]. These outcomes can also help to support wider community impact; for example, participant advocacy efforts have led community stakeholders to explore and learn from best practices applied in other cities, to mitigate barriers to healthy behavior in their own communities [25].

### 3.4. Step 4. Change

Step 4 research outcomes can directly relate to important health equity indicators, such as short- and long-term changes in social/environmental conditions, or formation of local groups of citizen scientists and stakeholders dedicated to advocacy strategies toward such changes [24,36]. Intended and unintended impacts of OV method activities can occur over time [35] and be identified by using ripple effect mapping techniques [37]. Individual and social-level benefits at this stage have included fostering self-confidence and enhanced social capital and civic engagement [36].

Capacity-building to cultivate skills in strategic communication and advocacy approaches among citizen scientists has been identified as an important process outcome at this stage [24]. Extended timelines of communication among citizen scientists, stakeholders, and researchers beyond the anticipated conclusion of a project can represent connections and group capacity for continued work to address long-term health equity issues. Form and frequency of communication at this step varies; one example featured standing, monthly meetings for continued planning, and evaluation [36].

Across Step 4 community outcomes, some citizen scientists have disseminated project findings to raise awareness among family members, neighbors, and local organizations, whereas others have formed advocacy teams to share concerns and issues with policy makers and have implemented school-based, peer-to-peer training activities [24,26,29]. Participants and stakeholders have strengthened and sustained local programming based on findings and relationships catalyzed throughout the project by using local policy seminars to advocate for benefits to the community [36], introduced web-based mobile applications to continue citizen science efforts for health promotion [25], and sought additional funding and resource support to maintain positive change within schools [31].

## 4. Hallmark Features of the OV Method

This article has focused on the opportunities for relevant health equity research, processes, and community outcomes at each step of the OV method and introduced a conceptual model with step-by-step evidence-based examples of multi-level applications. This information can be used by a range of action-focused, multidisciplinary teams to plan projects blending community engaged participatory action and citizen science strategies. Below, hallmark characteristics of the OV method are identified, followed by current innovations in using the method and future directions.

### 4.1. Multifaceted Participatory Practices

The OV method uses multiple participatory strategies to engage citizen scientists in individual and group data collection and interpretation. In this way, the method yields relevant findings that are deeply embedded in everyday realities of community members. In addition, the process itself can deliver transformative and activating experiences for individuals to both improve understanding of their social and environmental surroundings, and support opportunities for community members to begin their own journey of activism for health equity. The intended overall aim is for research and community partners to partake as co-learners as citizen scientists are co-researchers. Below we discuss the deeper functions of participatory strategies applied to accomplish Steps 1 and 2.

Discovery Tool walks (Step 1) are undertaken by citizen scientists in their local environments. The power of the walk has been embraced by many traditions as a qualitative methodology, including the “go-along” [38,39] and walking interview in ethnographic research [40]. In OV projects, gathering narrative-laden data while walking may be more engaging and lead to different responses than sit-down [41]; *Our Voice New Orleans publication forthcoming in this special issue*. While some OV projects feature citizen scientists walking alone or in small groups for Step 1, other projects pair researchers or community stakeholders with citizen scientists. For researchers or community stakeholders, walking along with citizen scientists in their local environments can deliver unparalleled insights into daily lived experiences, and can simultaneously build trust by positioning the citizen scientist in the role of “local expert” while the researcher or stakeholder assumes the role of “listener and learner”.

The function of the Discovery Tool app may also bear resemblance to “counter-mapping”, a technique that appropriates top-down, technical maps to generate bottom-up alternatives maps [42]. The OV method allows for individual-level (and later, group-level) engagement with images of their material surroundings, which can deepen both citizen scientists’ and researchers’ understandings of the relationships between people and their environments.

The participatory data review and interpretation process (Step 2) brings citizen scientists together as a group to thematically organize and assign meaning to their collective multimedia data. The group interactions and multivocal narratives included in participatory group processes have been acknowledged as a useful strategy in uncovering subjugated knowledge from the vantage of historically underrepresented populations [43]. Step 2 group processes are intended for universal application and tailoring across projects, and generally build from methods of community forums, listening sessions, and interpretive focus groups, which engage community members, who share common experiences due to geographic, socioeconomic, or other characteristics, to analyze data relating to their community and discuss various aspects [44,45].

This approach moves beyond researcher-based interpretation of citizen scientists’ thoughts, feelings, and experiences and positions local knowledge and perspective at the center of the data analysis process. Step 2 deepens the participatory function by engaging the same citizen scientists who collected the data to also participate as analysts and interpreters of their own collective data. In doing so, this step can be described as building on conceptual approaches such as “feminist-infused” participatory research [46] to address what Dodson and colleagues refer to as the “ethics of interpreting data from other people’s lives” [44] (p. 175).

### 4.2. Adaptability and Accessibility

The OV method has been informed by a range of behavioral, social, and environmental theories of change [13]. The adaptable nature of this method allows researchers and practitioners across disciplines, such as medical anthropology and human geography, to layer new theories and perspectives on the method. For instance, current research deploying the OV method is utilizing Doreen Massey’s [47] relational approach to place/settings to better understand the social worlds of people who use drugs and seek harm-reduction services. In addition to facilitating a diversity of partnerships, OV projects across the globe have engaged citizen scientists across diverse age groups and racial, ethnic, and socioeconomic backgrounds [16]. People who may not otherwise participate in health research can determine their own level of participation, from planning study design and methods, to partaking in a Discovery Tool walk to collect one or more images and comments, to spearheading action beyond the life of an “official” project.

Although the method intentionally allows for adaptability and accessibility, the structure and four-step process emphasize systematic data collectio1n, analysis, and community-driven solutions for change [21]. The OV initiative provides step-by-step training via a community engagement and research support team, technology, and tools to systematically support project development to dissemination. These supports include the following: the Discovery Tool mobile application, password-protected access to a secure web platform for processing data, individual and community-level maps in the form of project reports to review data, an OV implementation toolkit, and administrative dashboard technology [16].

### 4.3. Iterative Process with Potential for Cumulative Results

As an iterative process, each OV step promotes learning, synthesis, and reflexivity around a health issue through returning to these critical inquiries at each step. The activate (Step 3) and change (Step 4) steps of the OV method provide a robust structure for engaging citizen scientists in a way that can be context-specific and adaptable to the local needs and concerns of these individuals. Utilizing participatory processes, including discussion groups and community workshops, citizen scientists and community stakeholders can develop iterative applications to generate data-driven action plans and form an impetus for change.

**Figure 2:**
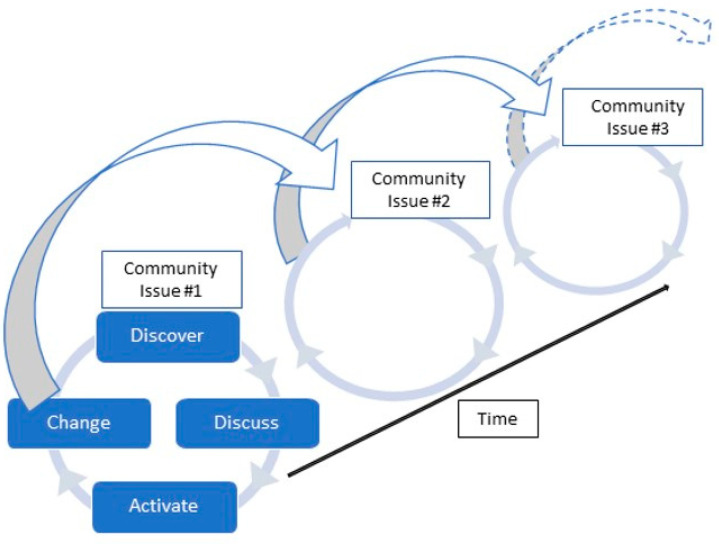
The OV method strategy for cumulative, cyclical community impact over time.

In this way, learning builds on the outcome of the previous step as the starting point for each next iteration. In a departure from a standard, left-to-right pathway, we visualize the potential cumulative function of the OV method within a community setting as an overlapping, cyclical process evolving or morphing over time (Figure 2). This visualization conveys how the OV method can deliver a strategy for committed, community-engaged research that maintains the capacity and mission to address distinct, yet thematically linked health equity issues over time. This approach stands in contrast to “snapshot” research projects which may uncover complex issues yet lack the inherent iterative structure to move forward in addressing the next logical issue(s). In this way, cumulative positive effects may be yielded at the research, process, and community levels across time as capacity building, empowerment, and awareness are raised across community members and leadership structures.

An excellent example of this iterative, forward-moving process that OV and similar participatory action research models can unleash is demonstrated in an OV project centered originally on access to healthful food choices among lower-income populations of older adults in San Mateo County, CA [24]. Although the original short-term (three-month) project yielded useful information about the food-related needs, particularly in relation to transportation and similar issues, of participating older adults, the activation and enablement of the citizen scientists did not end with the end of this project. In fact, at six months, citizen scientists took part in further advocacy training to form a senior advocacy team, communicating the concerns of older adults with policymakers at both local and state levels. At 24 months, the senior advocacy team had also partnered with a local elementary school, fostering changes to promote pedestrian safety. These changes in turn held implications for individuals accessing food stores by public transport or foot, promoting safer environments.

## 5. Innovations in Current OV Projects

Recently, innovations across OV projects have given rise to potential methodological advancements, critical questions of collective identity, and new process outcomes.

Methodological advancements in current OV projects highlight the potential for use in new settings and questions in health research. For instance, researchers are exploring ripple effects mapping [17], integrating data from sensors [48], and layering data from big data sources like Google Street view (see visibleghosts.com) and state level data. Current research questions address global health equity topics such as gender equality (e.g., developing strategies for increased sport and physical activity participation by girls and women in Peru) and food security (e.g., utilizing the OV method as a decolonizing methodology to support family-based nutrition in remote Indigenous communities).

As OV increasingly integrates big data sources and visualizations, researchers using the method have sought to adopt an anti-essentialist approach to collective identity [49]. Corburn [50], elaborates on this approach and suggests, “no person has a single, easily stated, unitary identity and that no absolute ‘truth’ exists from any one perspective” (p. 421). Engaging people from underrepresented communities as “citizen scientists” can highlight their counter-expertise in the face of overly deterministic narratives and open up space to express alternative stories. Strategies have included piloting a “post-journey” interview for citizen scientists to express their experience of sharing their story on a walk as a means for deeper discussion. Researchers have also explored ways to share and respond to rich stories collected during OV studies in creative ways, for example, through using an online, object-based storytelling exhibit, interactive community-level maps, and response art pieces from amateur cartographers who charted maps of their own neighborhoods (www.visibleghosts.com). Although OV facilitators are trained to honor diverse and outlier perspectives, larger issues within co-production and participatory action research remain. Namely, different versions of the same place and situation within a heterogeneous group can complicate a collective narrative, consensus, and the boundaries of a community. An emerging area of development considers, even as the group move toward prioritization, strategies to ensure all voices are valued, transparency, and underscore where tensions may lie.

As the OV initiative has reflected on powerful aspects of method, an additional, optional step in the method, entitled “Imagine”, is under consideration. This step would enhance the method by allowing citizen scientists and researchers to envision the possible changes emerging from the Discover and Discuss steps by developing compelling visualizations of the community-generated data using the emergent technology of virtual reality and similar state-of-the-art platforms. Such visualizations can in turn be a powerful tool for activating community members, stakeholders, and decision makers in making relevant changes reflected in the final step.

## 6. Limitations and Future Directions

Limitations and avenues for further strengthening the OV method exist. Engaging small groups of citizen scientists can present limitations in terms of scalability and representativeness of outcomes, which may overlook populations not engaged, or miss wider health concerns [21,23,24]. Additionally, many OV projects lack resources to continue to identify and evaluate actions and solutions implemented over the long term once a project has been completed [33,36]. Future OV projects aim to build pathways that extend the capacity of all participants engaged (researchers, community partners, and citizen scientists) to activate short- and long-term ripple effects, paired with the planning and resources to monitor long-term outcomes. This includes ensuring engagement of diverse populations and settings to strengthen the representativeness of outcomes [32]. An increasing number of projects are also utilizing more rigorous research design methods (e.g., randomized controlled experiments, natural experiments) to expand scientific advances and contributions in this area [13,51].

Future OV projects will aim to extend the content of areas and topics explored, such as incorporating the relevance of community-based perspectives for infectious disease and health equity. The emergence of the global COVID-19 pandemic has highlighted the importance of considering more chronic forms of health impacts which may require further research to understand how these impacts occur for different communities. Similarly, the OV method will continue to be utilized to engage community members in understanding context-specific and relevant concerns and solutions that can have real-world impacts when scaled up to decision-making levels. Engaging decision makers should be further considered, particularly as such roles can provide economic and social resources and support sustainability for changes that are implemented.

Further engagement of decision makers and community stakeholders in the four steps of the OV method could give rise to an understanding of how decisions are made for local communities. This may also provide avenues for understanding concerns at the decision-making level, such as dependence on higher-level sources of support and budgetary constraints that impact community-driven solutions [31].

## 7. Conclusions

We have presented an approach that includes citizen science methods combined with comprehensive participatory action research methods. As exemplified by the OV method, fusing these perspectives can lead to powerful outcomes in health promotion efforts. Positioning community members, stakeholders, and researchers as co-producers of knowledge creates fertile ground for both knowledge creation and community transformation [52]. Marrying such a community-based participatory philosophy with innovative citizen science methods—supported by systematic data collection, visualization, and delivery technologies—provides a powerful toolkit for tackling our most pressing health equity challenges.

## Figures and Tables

**Table 1:** The OV four-step method across research, process, and community outcomes.

*Our Voice* Method Steps	Outcome Category		
	Research (Building Knowledge)	Process (Building Capacity)	Community (Building Health)
Step 1: Discover	Co-produce individual citizen scientist understanding and embodied experiences in selected settings	Establish equitable distribution of power across partnership as citizen scientists control data collection	Assemble a resource of citizen scientist collected data that can be used in further advocacy efforts
	Understand acceptability of technology-based methods and citizen science approach in different populations	Enhance awareness among citizen scientists about influence of environment on health	Experience benefits of data gathering, e.g., collective engagement and contributions to science and the community
		Help to build self-efficacy for using digital tools	Experience therapeutic benefits of participating in a research walk, e.g., improved mental and/or physical health
Step 2: Discuss	Co-generate understanding of group perspectives and interpretations of one’s own experiences in selected setting	Facilitate awareness of collective issues in selected setting	Catalyze new relationships across citizen scientists and community stakeholders
	Identify needs or barriers and sources of strength for health-focused topic	Foster trust across partners through transparent research process Strengthen social connections across community members	Assign priority for action based on community values
	Characterize points of consensus and tension for health-focused topic		Generate collective solution-building using diverse, inclusive process
			Build group momentum for action toward change
			Identify trusted leaders/stakeholders within the community
Step 3: Activate	Identify appropriate research directions to match community priorities for inquiry and improvement	Initiate open lines for equitable communication within partnership about change, who can contribute, and how	Encourage exchange of local knowledge and desired actions between service users, providers and community members
		Deliver advocacy training to citizen scientists to learn new skills and cultivate empowerment	Foster use of data-driven decisions among community stakeholders
			Reinforce support for or inform changes to existing plans, programs, or policies
Step 4: Change	Complete short- and long-term improvements in social and/or environmental conditions, which impact health indicators	Further train citizen scientists in topics such as strategic communication, additional advocacy strategies, and ripple effects activities	Disseminate findings by citizen scientists across local networks
	Intended and unintended impacts across time (ripple effects)	Extend partnership timeline to address long-term issues	Form groups to advocate change to decision-makers
			Bolster or sustain existing programming
			Form coalitions across citizen scientists and stakeholders to sustain change
